# Editorial: Mixotrophic, Secondary Heterotrophic, and Parasitic Algae

**DOI:** 10.3389/fpls.2021.798555

**Published:** 2021-11-25

**Authors:** Miroslav Oborník, Richard G. Dorrell, Denis V. Tikhonenkov

**Affiliations:** ^1^Biology Centre, Institute of Parasitology, Czech Academy of Sciences, České Budějovice, Czechia; ^2^Faculty of Science, University of South Bohemia, České Budějovice, Czechia; ^3^Département de Biologie, Institut de Biologie de l'ENS (IBENS), École Normale Supérieure, CNRS, INSERM, Université PSL, Paris, France; ^4^Papanin Institute for Biology of Inland Waters, Russian Academy of Sciences, Borok, Russia; ^5^AquaBioSafe Laboratory, University of Tyumen, Tyumen, Russia

**Keywords:** photosynthesis, loss, plastid, endosymbiosis, evolution

Two biochemical processes built on electron transport chains stand behind the success of life on Earth: photosynthesis, which transforms the energy of sunlight into the energy of chemical bonds in primary metabolites, and heterotrophic respiration of this organic energy. This bioenergetic cycle has enabled the evolution of the extraordinary living complexity of the planet.

Photosynthesis originated in eukaryotes *via* several independent acquisitions of the photosynthetic machinery through the endosymbioses of phototrophic symbionts, giving rise to “plastids” (also referred to as chloroplasts). The symbionts were either cyanobacteria in primary endosymbioses or eukaryotic algae in complex endosymbioses (Figure 1) (Keeling, 2013; Archibald, 2015; Oborník, 2019). The repeated endosymbiotic acquisition of plastids has led to a rich diversity of photosynthetic plants and eukaryotic algae found in most environments on Earth with access to light.

**Figure 1:**
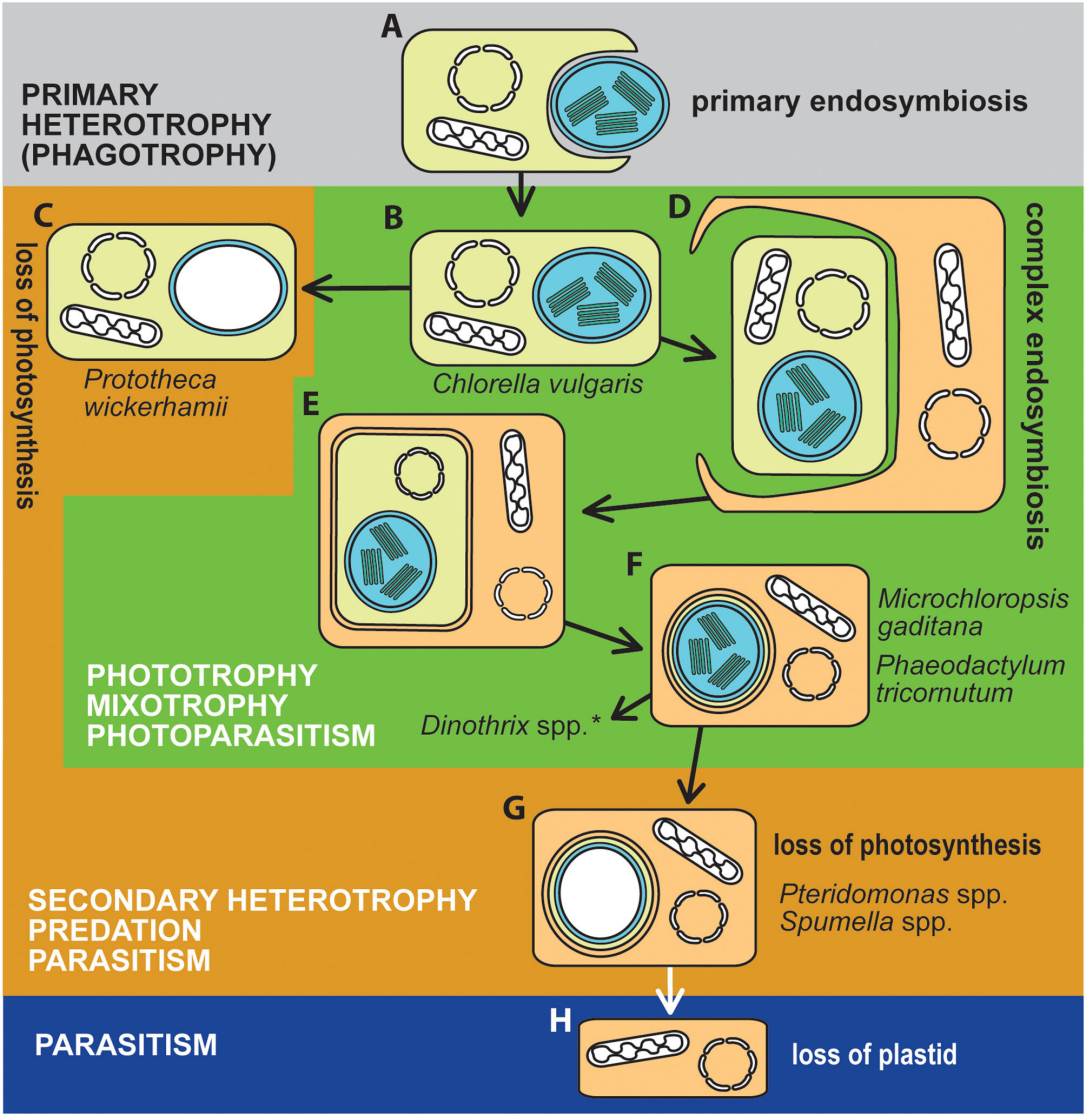
The trophic evolution of algae. Algae became phototrophic through endosymbioses with cyanobacteria (**A,B**; in Archaeplastida and *Paulinella*), or eukaryotic algae (**D,E**; Cryptophyta a Chlorarachniophyta, both retaining the nucleomorph; **F**, algae with complex plastids surrounded by three membranes, such as Euglenophyta and Dinophyta, or algae with four membranes plastid envelops, Ochrophyta, Apicomplexa including chromerids, Haptophyta, and Dinophyta with higher-order plastids). Photoautotrophic algae with primary plastids **(B)** lost photosynthesis in some lineages (e.g., *Helicosporidium, Prototheca, Polytomella*), becoming secondarily heterotrophic **(C)**. Photosynthesis was more frequently lost from algae with complex plastids, particularly from alveolates **(G)**. Plastids were further completely lost from apicomplexan (*Cryptosporidium*) and dinoflagellate (*Hematodinium*) **(H)** parasitic lineages, with essential compounds typically produced by plastids, instead scavenged from the host. Particular species studied in the Research Topic are indicated in the figure. ^*^Dinothrix spp. is a dinotom with a much more complex cell structure: it hosts the diatom endosymbiont, which still retains the nucleus, mitochondrion, and the diatom complex plastid. In addition to that, dinotoms contain a relic non-photosynthetic dinoflagellate plastid.

Despite the advantage of phototrophy, many lineages retain heterotrophic abilities resulting in mixotrophy. Mixotrophy is a combination of photosynthesis and heterotrophic lifestyles *via* osmo-heterotrophy, phagotrophic predation, or even parasitism (Oborník, 2020). Mixotrophic organisms perform heterotrophy to acquire organic carbon or scarce and growth-limiting nutrients (e.g., nitrogen) from their external prey or host. Mixotrophy may re-evolve in species that were previously only phototrophs, with beautiful examples of carnivory (Venus flytraps, sundews, pitcher plants) celebrated within the plants (Fukushima et al., 2017; Fleischmann et al., 2018; Palfalvi et al., 2020). A further form of mixotrophy is found within some obligately heterotrophic eukaryotes (e.g., *Paramecium* ciliates, lichens, and corals), which support long-term photosynthetic symbionts as a source of primary metabolites and may provide us with clues into the cellular innovations underpinning the endosymbiotic acquisition of the chloroplast (Johnson et al., 2006; Dorrell and Howe, 2012; Stoecker et al., 2017).

Despite its benefits to primary production, photosynthesis is not necessarily retained in all plastid-containing organisms forever. Mixotrophs are particularly prone to lose photosynthesis, which has been documented for many lineages of algae with primary and complex plastids. “Algae” therefore encompass a wide range of trophic modes, ranging from pure phototrophs, through mixotrophs with different dependences on external carbon, to obligatory phagotrophs and parasites unable to live without a host. Although it is an extremely rare event, the plastid can be completely lost. Such cases are found exclusively among parasites; e.g., in the apicomplexan parasites *Gregarina niphandroides* (Toso and Omoto, 2007) and *Cryptosporidium parvum* (Zhu et al., 2000), and the parasitic dinoflagellate *Hematodinium* sp. (Gornik et al., 2015) (Figure 1). In other obligately heterotrophic algae and plants, the plastid may be retained as a membrane-bound compartment with associated metabolic functions but no endogenous DNA (Molina et al., 2014; Smith and Lee, 2014; Dorrell et al., 2019).

This Research Topic presents eight articles exploring the diverse taxonomy and metabolic potential of mixotrophic, secondary heterotrophic, and parasitic algae. Three articles focus on algae with non-photosynthetic plastids (Bakula et al.; Kayama et al.; Kim et al.). A range of metabolic functions have been assigned to non-photosynthetic plastids across the tree of life (Hadariová et al., 2018). For example, the apicoplast, the relic plastid in apicomplexan parasites, synthesizes heme, isoprenoids, fatty acids, and Fe-S clusters (Ralph et al., 2004), whereas the osmo-heterotrophic euglenophyte *Euglena longa* and the dictyochophyte *Pteridomonas danica* retain a plastid-encoded gene for the large subunit of RuBisCo (*rbc*L), which may modulate the redox balance of the cell via a linearized Calvin-Benson pathway (Sekiguchi et al., 2002; Füssy et al., 2020).

The articles presented highlight the diversity of parasitic plastid genomes, even between closely related species within individual genera. Kayama et al. present plastomes from other *Pteridomonas* species to *P. danica* that lack the *rbc*L gene and encode only housekeeping genes; and present transcriptomic evidence that the sequenced *Pteridomonas* spp. plastids are responsible for heme synthesis, glycolysis, and pentose phosphate pathways but do not perform Fe-S cluster assembly (Kayama et al.). Bakuła et al. describe the plastid and mitochondrial genomes of the non-photosynthetic green alga *Prototheca wickerhamii*, a causative agent of human protothecosis (Lass-Flörl and Mayr, 2007), alongside related non-photosynthetic species. The analysis suggests that independent losses of photosynthesis may underpin different plastid genome contents across *Prototheca* (Bakuła et al.). Finally, Kim et al. sequence and analyse the genomes of non-photosynthetic plastids from *Spumella*-like chrysophytes, tiny heterotrophic bacterivorous flagellates. Similar to the sequenced plastid genome of the related *Spumella* NIES-1846, the genomes contain housekeeping genes but still show important lineage-specific differences (Dorrell et al., 2019; Kim et al.). The diverse trends of non-photosynthetic metabolism uncovered in all three studies may reflect the phylogenetic diversity of each lineage, with both *Prototheca* and *Spumella* known to be polyphyletic (Bakuła et al.; Dorrell et al., 2019).

Three further articles focus on mixotrophic algae (Dal Bo et al.; Dani et al.; Villanova et al.). Dal Bo et al. investigate the mixotrophic growth of *Microchloropsis gaditana* on different organic compounds, showing through a transcription activator-like effector nuclease (TALE-N) knockout of the mitochondrial alternative oxidase AOX1 that this mixotrophic growth depends primarily on mitochondrial respiration rather than photosynthetic activity (Dal Bo et al.). Dani et al. report that the mixotrophic species *Chlorella vulgaris* can emit isoprene in phototrophic conditions under light and also when grown as a pure heterotroph on glucose in complete darkness. The dark-associated function of isoprene is unknown and may contribute to anomalies in estimates of oceanic isoprene concentrations (Dani et al.). Villanova et al. present an experimental model of biomass production in the diatom *Phaeodactylum tricornutum*, to optimize the composition of cultivation media and light intensity to boost biomass quantity and quality. These approaches aim to overcome growth-limiting effects of nitrogen starvation, which is usually used to induce algal lipid production (Villanova et al.). All three articles underline that even species typically thought of as “photosynthetic” may engage in mixotrophy in the wild, and that this potential may be harnessed to improve biomass production for aquacultures and sustainable biotechnologies (Lowrey et al., 2015; Saad et al., 2019).

The final two articles focus on algal phylogeny and taxonomy (Yamada et al.; Jeong et al.): Yamada et al. investigate the phylogenetic positions of the dinotoms (dinoflagellates with diatom endosymbionts) *Dinothrix paradoxa* and *Gymnodinium quadrilobatum*. The authors show through sequencing and analysis of 18S rRNA and *rbc*L genes that these species are close relatives of another dinotom, *Galeidinium rugatum*, despite their distinctive morphologies and life cycles, with flagella-lacking cells as the predominant stage, and isolate and formally describe two new dinotom species, *Dinothrix phymatodea* and *Dinothrix pseudoparadoxa* (Yamada et al.). Finally, Jeong et al. show unprecedented insights into the phylogeny and diversity of *Spumella*-like chrysophytes by the use of nuclear rDNA data, revealing high molecular diversity despite morphologically convergent forms suitable for heterotrophy (Jeong et al.). Both studies underline the importance of molecular methods, alongside classical isolation and taxonomy, for untangling the complicated origins and diversifications of heterotrophic algae.

The collection of articles in the Research Topic illustrates the frequent losses of photosynthesis across the eukaryotes; and what molecular innovations allow algae to live as mixotrophs, heterotrophs, and parasites. The metabolic functions associated with these transitions transform our understanding of the roles of algae in supporting the planetary ecosystem and in the evolutionary plasticity of eukaryotes. Anything that can be used by life is used.

## Author Contributions

MO wrote the first draft of the manuscript. All authors contributed to the article and approved the submitted version.

## Funding

MO acknowledges funding by the Czech Science Foundation (21-03224S) and ERDF/ESF (CZ.02.1.01 /0.0/0.0/16_019/0000759). RD acknowledges a CNRS Momentum Fellowship, and an ANR JCJC Grant (PanArctica, ANR-21-CE02-0014-01). DT acknowledges funding by the Tyumen Oblast Government [project No. 89-DON (2)] and the Russian Foundation for Basic Research (20-04-00583).

## Conflict of Interest

The authors declare that the research was conducted in the absence of any commercial or financial relationships that could be construed as a potential conflict of interest.

## Publisher's Note

All claims expressed in this article are solely those of the authors and do not necessarily represent those of their affiliated organizations, or those of the publisher, the editors and the reviewers. Any product that may be evaluated in this article, or claim that may be made by its manufacturer, is not guaranteed or endorsed by the publisher.
